# Hallazgo incidental de una hemoglobina rara: hemoglobina Bari en el noreste de España

**DOI:** 10.1515/almed-2023-0070

**Published:** 2023-07-25

**Authors:** Raquel Lahoz Alonso, Naiara Romero Sánchez, Ruth González Sánchez, Antonia Escobar Medina, Aurora M. López Martos, Marta Domínguez García, David Beneitez Pastor, Montserrat Prieto Grueso, Adoración Blanco Álvarez, Susana Urban Giralt, Patricia Esteve Alcalde

**Affiliations:** Servicio de Análisis Clínicos, Hospital Ernest Lluch, Calatayud, Zaragoza, España; Atención Primaria, Centro de Salud Daroca, Daroca, Zaragoza, España; Unidad de Eritropatología, Servicio de Hematología, Hospital Universitari Vall d’Hebron, Barcelona, España; Técnico Superior Sanitario de Laboratorio Clínico y Biomédico, Unidad de Eritropatología, Laboratorios Clínicos Vall d’Hebron, Hospital Universitari Vall d’Hebron, Barcelona, España; Unidad de Genética Molecular Hematológica, Servicio de Hematología, Hospital Universitari Vall d’Hebron, Barcelona, España; Técnico Superior Sanitario de Laboratorio Clínico y Biomédico, Unidad de Genética Molecular Hematológica, Laboratorios Clínicos Vall d’Hebron, Hospital Universitari Vall d’Hebron, Barcelona, España

**Keywords:** HbA_1c_, hemoglobina Bari, HPLC

## Abstract

**Objetivos:**

La cromatografía líquida de alta resolución (HPLC) es una de las técnicas empleadas para determinar la hemoglobina glicosilada (HbA_1c_) y el método de elección para el cribado de hemoglobinopatías estructurales. El objetivo de este caso es mostrar cómo en un análisis de rutina de HbA_1c_ es posible encontrar incidentalmente una hemoglobinopatía.

**Caso clínico:**

En un análisis clínico rutinario, se observó un valor anormal de hemoglobina A_2_ (HbA_2_) en el análisis de HbA_1c_ mediante HPLC, realizado con el analizador ADAMS™ A1c HA-8180T. Ante la sospecha de la presencia de una hemoglobinopatía, se amplió el estudio a posibles variantes mediante electroforesis y HPLC, empleando los analizadores Hydrasys y Variant II, respectivamente. Dado que no se pudo identificar con ninguno de los métodos tradicionales, se realizó también un estudio genético mediante secuenciación Sanger. El paciente presentaba niveles bajos de HbA_2_ (1.3%) y una variante del 24,9% con un tiempo de retención de 1.95 minutos, compatible con una variante de la cadena de alfa-globina. En el estudio genético, se detectó la variante patogénica c.138C>G en el gen *HBA2* en heterocigosis, causando la expresión de la hemoglobinopatía conocida como hemoglobina Bari.

**Conclusiones:**

El cribado inicial de hemoglobinopatías estructurales permite su identificación o suscita sospecha de su presencia, especialmente cuando se realiza junto al análisis de HbA_1c_, requiriendo posterior confirmación y diagnóstico con otras técnicas.

## Introducción

La determinación de hemoglobina glicada, también conocida como hemoglobina A_1c_ (HbA_1c_), está recomendada para el diagnóstico y seguimiento de diabetes mellitus, con un umbral de ≥6,5 % [1]. Esto se puede realizar mediante estudios enzimáticos, inmunológicos o de separación, como la cromatografía o la electroforesis.

La cromatografía líquida de alta resolución (HPLC) es el método de elección para el cribado de hemoglobinopatías estructurales y la cuantificación de hemoglobina A_2_ (HbA_2_) y hemoglobina fetal (HbF) [2].

La hemoglobina A (HbA) está compuesta por cuatro subunidades, dos cadenas alfa codificadas por los genes del cromosoma 16 (Hemoglobina Alfa 1 (*HBA1*) y Hemoglobina Alfa 2 (*HBA* *2*)) y dos cadenas beta codificadas por un gen del cromosoma 11 (Hemoglobina Subunidad Beta (*HBB*)). Cada uno de estos genes puede presentar variantes genéticas. Se estima que el 7 % de la población presenta una variante de HbA. Las manifestaciones clínicas de estas variantes son heterogéneas, desde la anemia y la reticulocitosis a la ausencia total de signos clínicos [3, 4]. Una vez identificada una hemoglobinopatía, se recomienda la realización de pruebas genéticas moleculares para hallar la mutación responsable [2].

Existen sistemas automatizados para el análisis de HbF, HbA_2_, y HbA_0_, que alertan sobre la presencia de valores pico anormales. La ventaja de estos ensayos es que realizan un análisis de alto rendimiento de HbA_1c_, con el valor añadido de detectar la presencia de hemoglobinopatías.

Presentamos el caso de un individuo clínicamente sano, con un valor bajo de HbA_2_ causado por una variante de la hemoglobina de origen genético confirmado.

## Caso clínico

Varón de 40 años de edad sin los síntomas habituales de diabetes, originario de una región al noreste de España (Zaragoza), sin ningún antecedente médico de interés, a quien se le realizó un análisis de sangre de control en atención primaria, como medida preventiva. Se obtuvieron muestras de sangre total en tubos con anticoagulante EDTA K2 (Vacutainer™ Becton-Dickinson, Rutherford, NJ, USA).

El ensayo HbA_1c_ se realizó en el analizador ADAMS™ A1c HA-8180T (ARKRAY, Inc., Kyoto, Japón), un sistema de cromatografía líquida de alta resolución diseñado para separar y cuantificar la HbA_1c_, así como para detectar las variantes HbA_2_ y HbF de la hemoglobina. Previamente al análisis, se analizaron varios controles internos, con el fin de determinar los diferentes niveles de HbA_1c_, HbA_2_ y HbF y verificar su correcto funcionamiento.

**Figura 1:**
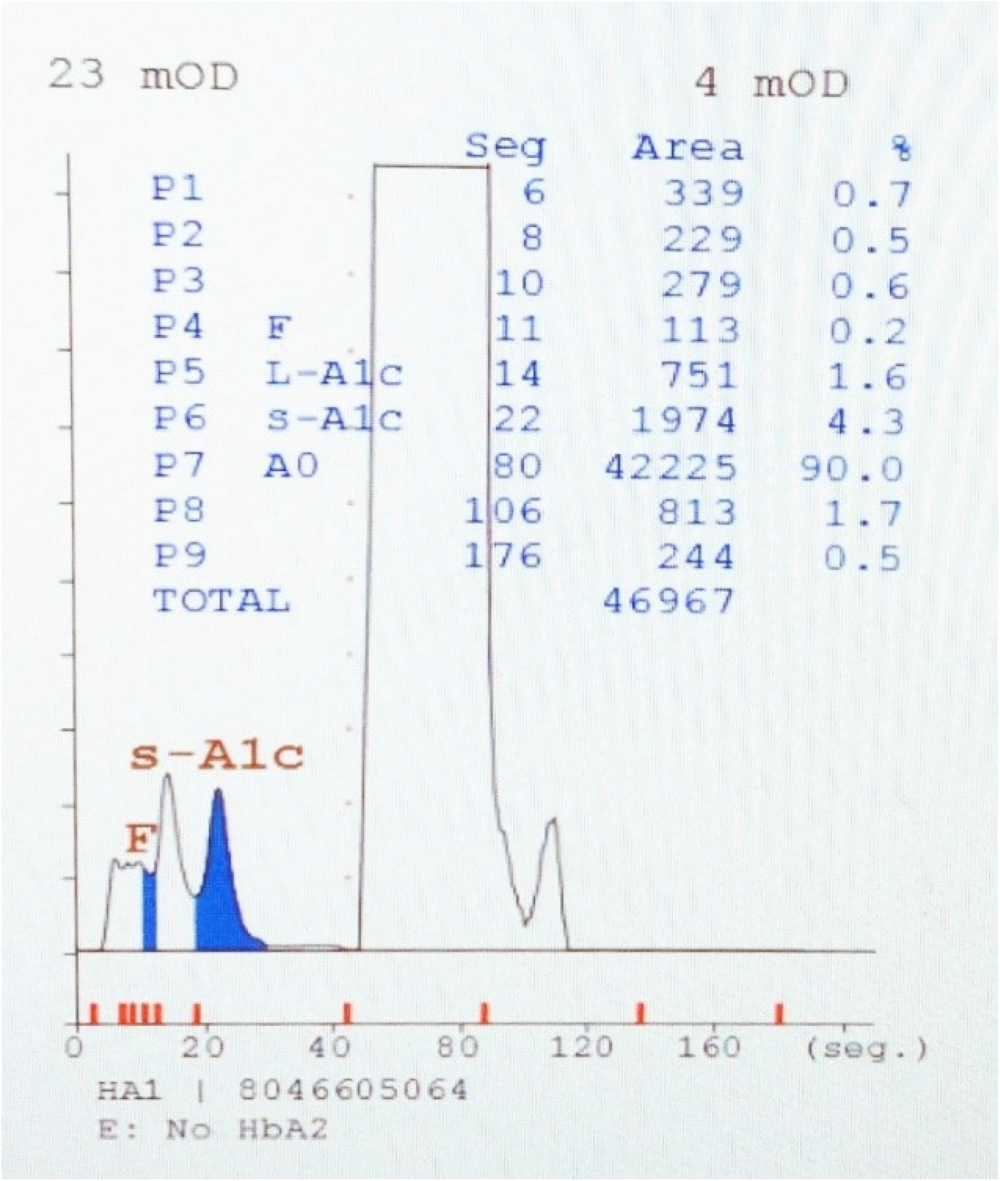
Resultados obtenidos con el analizador Arkray ADAMS A1C HA-8180T.

Mientras que el nivel de HbA_1c_ del paciente era normal (4,5 %), no se pudo determinar el nivel de HbA_2_ (Figura 1). Los resultados del hemograma fueron normales.

**Figura 2:**
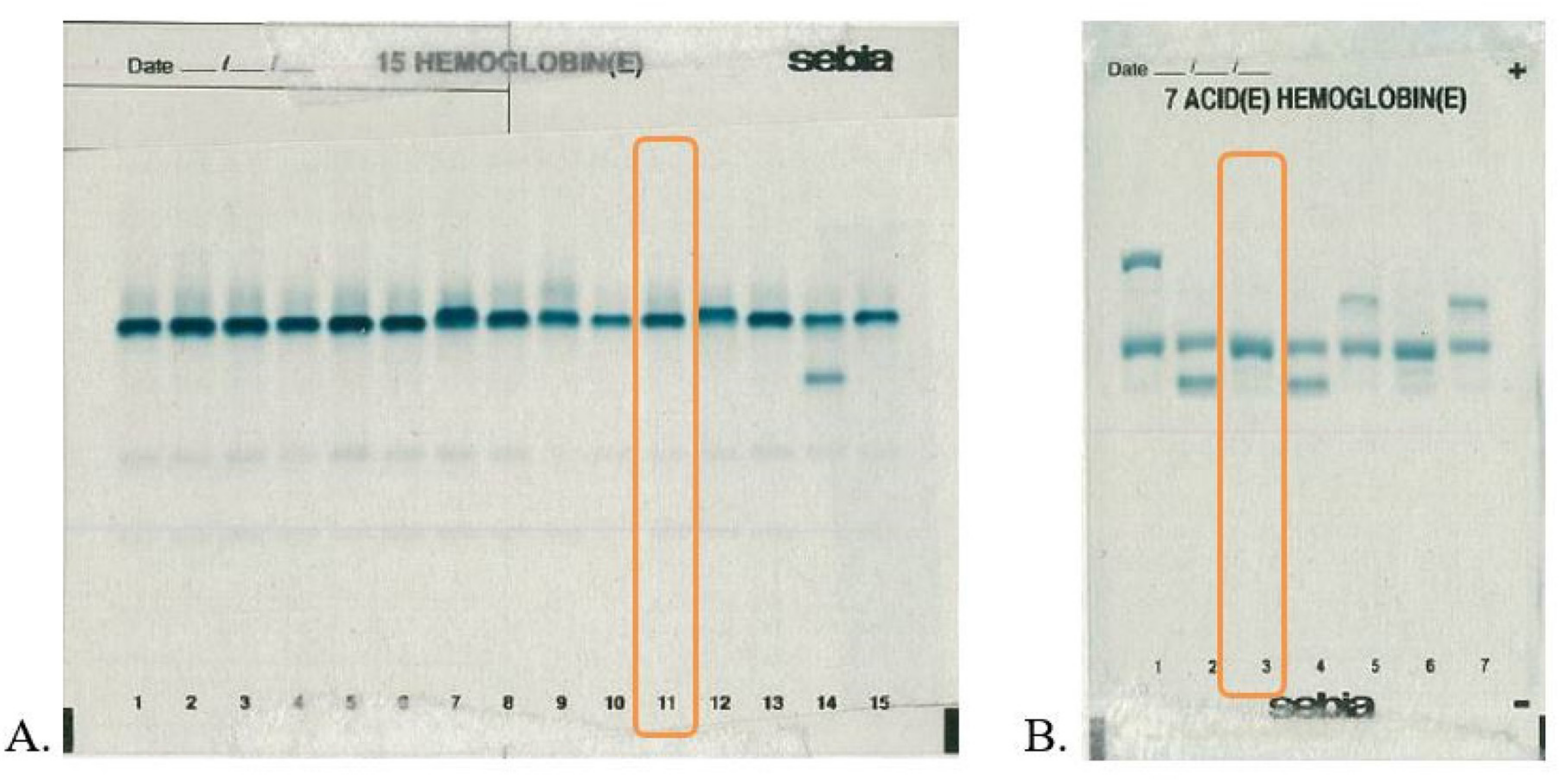
Estudio de eletroforesis. (A) a pH alcalino (canal 11) y (B) a pH ácido (canal 3) con el analizador Hydrasis.

A continuación, se realizó la electroforesis de hemoglobina con el analizador Hydrasys (Sebia Hispania^®^), para determinar la presencia de variantes de la hemoglobina. Cuando se sometieron las muestras a electroforesis a pH alcalino, estas mostraron un patrón de migración de hemoglobina normal. Un análisis adicional a pH ácido reveló una banda anormal que no se separaba de la HbA_1_ (Figura 2).

**Figura 3:**
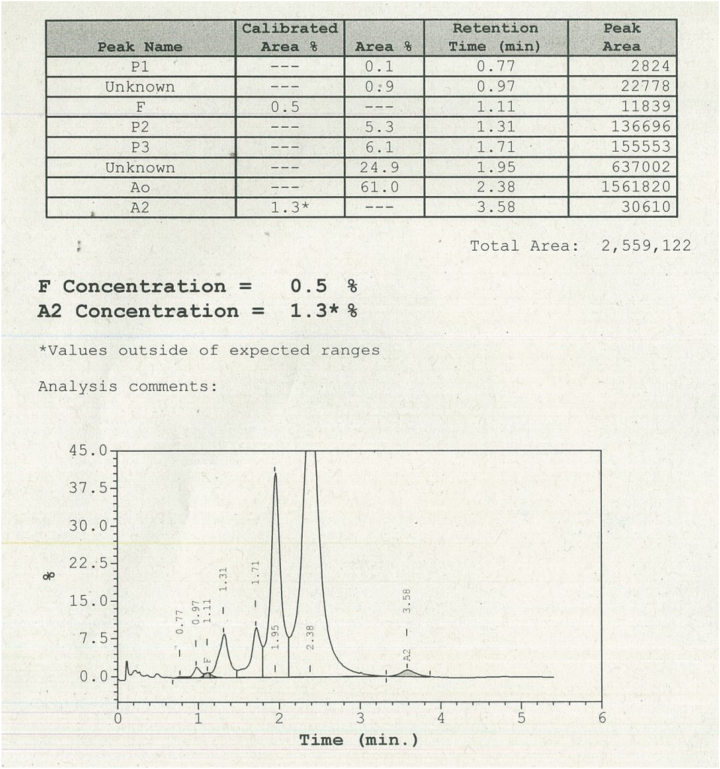
Resultados de HPLC otbenidos con el analizador Variant II.

Tras este análisis preliminar, la muestra se envió para su análisis mediante HPLC en el analizador Variant II (Bio-Rad^®^). El cromatograma mostró un pico con una fracción del 24,9 %, y un tiempo de retención de 1,95 minutos, compatible con una variante de la cadena alfa de globina (Figura 3).

Dada la presencia de un pico anómalo, se realizó el estudio genético. Se llevó a cabo la secuenciación Sanger de los genes *HBA1* y *HBA* *2*, estudiando las regiones exónicas y flanqueantes de los dos genes. Se identificó una variante patogénica en el exón 2 de *HBA* *2*, el gen que codifica la hemoglobina alfa 2, en heterocigosis. La variante de un solo nucleótido c.138C>G (citosina a guanina) en la posición de codificación 46 causa una sustitución de un solo aminoácido de histidina a glutamina. La variante estructural ha sido anteriormente identificada como hemoglobina Bari.

**Figura 4:**
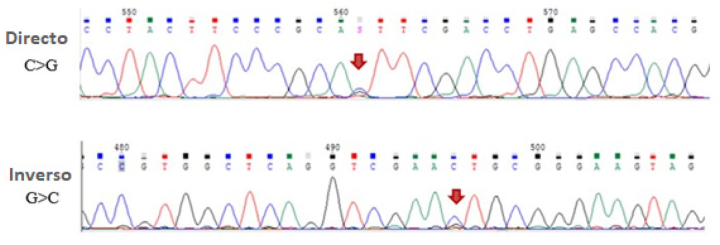
Mutación detectada en la secuenciación del gen *HBA*2.

En la Figura 4 se muestra la mutación en la secuencia del gen *HBA* *2*.

## Discusión

Ante la sospecha de la presencia de una hemoglobinopatía suscitada por el análisis de HbA_1c_, identificamos una variante estructural de las cadenas alfa de globina, conocida como hemoglobina Bari. Hasta donde nosotros sabemos, este es el segundo caso informado de esta variante. El gen implicado en este caso fue el gen *HBA* *2* *.* La hemoglobina Bari se descubrió y describió por primera vez en un hombre de 21 años procedente del sur de Italia (Calabria) [5]. Ambos pacientes presentaron niveles similares de la variante, del 20 % en el primer caso, y del 24,9 % en el caso aquí descrito.

Aunque la sustitución del aminoácido que se produce en esta variante implica un contacto distal con el grupo hemo, la molécula de la hemoglobina parecía estar completamente estable y con una funcionalidad normal, lo que explicaría el comportamiento similar al de la HbA en el análisis HPLC con el analizador Variant II, dado que esta eluye unos segundos antes. Además, los parámetros funcionales analizados en la muestra (P_50_ O_2_, cooperatividad y efecto Bohr) fueron similares a los de las muestras de control normales [5]. Estos hallazgos explican la ausencia de patología en ambos individuos.

Marinucci M. et al. observaron que la variante mostraba un comportamiento electroforético igual al de la HbA, aunque las cadenas de alfa globina anormales se movían más anódicamente que las normales a un pH de 6 [5]. En nuestro caso, ocurrió lo mismo, ya que la electroforesis a un pH ácido mostró una banda anormal que no se separaba de la hemoglobina A_1_, que migra de forma más anódica, tal como muestra la Figura 2.

El empleo de dos analizadores HPLC diferentes nos permitió identificar el valor pico anormal de hemoglobina, así como cuantificar la HbA_2_. La principal diferencia entre ambos es el tiempo de elución, siendo este de 3,5 minutos en el sistema ADAMS™ A1c HA-8180T y de 6,5 minutos en el Variant II. De este modo, el analizador Variant II, al procesar la muestra durante más tiempo, permitió separar correctamente de la HbA el pico anómalo de la variante hemoglobina Bari, y cuantificar el bajo valor de HbA_2_. En los heterocigotos para variantes estructurales de cadena alfa, los tres tipos de hemoglobinas del adulto se verán afectadas [2], por lo que el valor anormal de HbA_2_ podría no ser identificado y cuantificado correctamente, presentando un nivel más bajo de HbA_2_.

La prevalencia de cada hemoglobinopatía varía en las diferentes regiones del mundo, aunque estas son más comunes en las regiones mediterráneas y tropicales de África y Asia [6]. En nuestro caso, al igual que en el caso anterior [5], los dos individuos procedían de países mediterráneos. El hecho de que únicamente se haya informado de dos casos podría deberse a la ausencia de síntomas, por lo que su prevalencia podría estar subestimada. A pesar del carácter asintomático de esta patología, el asesoramiento genético es importante en pacientes en edad fértil [2]. Por ello, sería interesante ampliar el estudio a los familiares del paciente, aunque no disponemos de más datos por el momento, ya que el paciente ha rehusado realizarle el estudio a sus descendientes.

Las variantes de la hemoglobina pueden interferir en la cuantificación de la HbA_1c_, aunque la mayoría de los ensayos que se utilizan actualmente no se ven afectados por las variantes más comunes (S, C, D, E). Sin embargo, algunos analizadores ofrecen resultados discordantes [7]. En la práctica clínica, la presencia de una variante de la hemoglobina con valores extremos de HbA_1c_ (<4 % o >16 %) no concordantes con los niveles de glucosa debería suscitar sospechas [8]. Además de la presencia de variantes menos comunes que pueden interferir en la cuantificación de HbA_1c_ [8, 9], existen variantes dependientes de la raza o etnia o de otras patologías. Los resultados pueden estar alterados en casos asociados a un recambio elevado de glóbulos rojos (anemia de células falciformes, embarazo (segundo y tercer trimestre), deficiencia de glucosa-6-fosfato deshidrogenasa, hemodiálisis, pérdida de sangre reciente, transfusión o terapia con eritropoyetina), posparto, VIH tratado con ciertos inhibidores de la proteasa e inhibidores de la transcriptasa inversa y anemia por deficiencia de hierro [10]. Aun cuando nuestro paciente mostró niveles normales de glucosa y HbA_1c_, creemos que se podría haber subestimado la HbA_1c_ debido a la presencia de la variante de hemoglobina Bari, ya que obtuvimos un valor muy próximo al 4 %. No identificamos ninguna otra patología que pudiera haber ejercido como factor de confusión en la asociación entre los niveles de HbA_1c_ y de glucemia. Sería necesario realizar más estudios para confirmar este fenómeno.

En conclusión, cuando se realiza un análisis de HbA_1c_ mediante HPLC, es recomendable incluir el cribado de hemoglobinopatías estructurales, ya que esto permite su detección, especialmente en pacientes asintomáticos sin antecedentes de interés. En estos casos, sería necesario ampliar el estudio, con el fin de confirmar los resultados y establecer un diagnóstico definitivo. También hay que tener en cuenta que su presencia podría interferir en la determinación de HbA_1c_, por lo que es necesario analizar posibles discrepancias entre los valores de HbA_1c_ y de glucemia.

## Lecciones aprendidas

1. El cribado de hemoglobinopatías estructurales al realizar un estudio de HbA_1c_ mediante HPLC permite su detección.
2. La presencia de variantes de hemoglobina podría interferir en la determinación de HbA_1c_ mediante HPLC. En caso de discrepancia entre los niveles de HbA_1c_ y de glucemia es necesario realizar más estudios.
3. La hemoglobina Bari es una hemoglobinopatía estructural rara causada por una mutación en el gen *HBA* *2*, siendo esta asintomática en los portadores heterocigóticos de la variante.
